# Toward the “unravelling” of HIV: Host cell factors involved in HIV-1 core uncoating

**DOI:** 10.1371/journal.ppat.1007270

**Published:** 2018-10-04

**Authors:** Daniel J. Rawle, David Harrich

**Affiliations:** 1 Department of Cell and Molecular Biology, QIMR Berghofer Medical Research Institute, Herston, Australia; 2 School of Chemistry and Molecular Biosciences, University of Queensland, St. Lucia, Australia; Mount Sinai School of Medicine, UNITED STATES

## Introduction to HIV-1 uncoating: Down the rabbit hole

**Fig 1:**
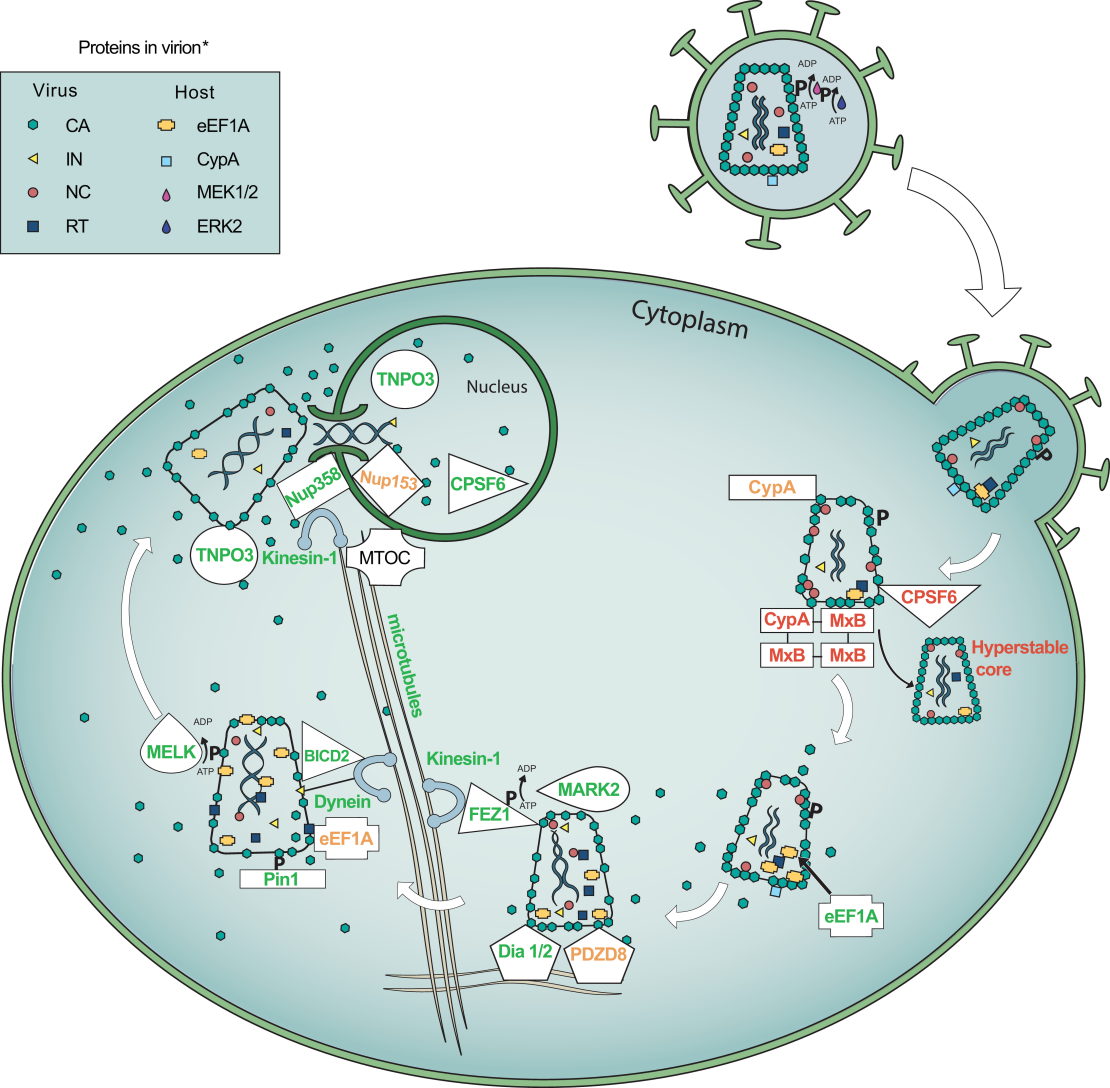
Host cell factors regulate HIV-1 uncoating. The HIV-1 virion, which contains the CypA, ERK2, and eEF1A host proteins that regulate uncoating, binds and fuses with the host cell membrane, and the core is released into the cytoplasm. PDZD8 and CypA binds CA to stabilize cores to promote infection; however, infection is inhibited if CypA associates with MxB, in which MxB oligomers bind CA to hyperstabilize cores. Cellular eEF1A interacts with HIV-1 RT and facilitates uncoating. Dia1 and Dia2 bind CA–NC complexes to facilitate uncoating likely by localized microtubules stabilization. Dynein interacts with the core via the BICD2 adapter protein or direct interaction with IN, and kinesin-1 interacts with the core via the FEZ1 (phosphorylated by MARK2) adapter protein. This is important for transport of the replication complexes through the cytoplasm to MTOCs at the nuclear periphery as well as uncoating. Pin1 binds CA phosphorylated by MEK1/2 activated ERK2 during virion maturation to facilitate uncoating. MELK also phosphorylates CA to promote uncoating. HIV-1 RT binds eEF1A to stabilize the RTC. The NPC proteins Nup358 and Nup153 help import the PIC, and Nup358 is relocalized to the cytoplasm by KIF5B, while Nup153 prolongs CA association with PICs in the nucleus. TNPO3 localizes CPSF6 to the nucleus to prevent CPSF6-dependent hyperstable cores, and TNPO3 may also help complete CA dissociation from replication complexes in the nucleus. This figure does not attempt to illustrate the spatiotemporal specifics of where and when host factors interact with the core and regulate CA uncoating. Green text: promotes uncoating for optimal kinetics. Orange text: delays uncoating for optimal kinetics. Red text: causes hyperstable cores and inhibits infection. Only viral and host proteins relevant in uncoating and discussed in this article are included in the figure. CA, capsid; CPSF6, cleavage and polyadenylation specificity factor 6; CypA, cyclophilin A; BICD2, bicaudal D2; Dia1/2, diaphanous-related formins 1 and 2; eEF1A, eukaryotic translation elongation factor 1A; ERK2, extracellular signal-regulated kinase 2; FEZ1, fasciculation and elongation factor zeta 1; IN, integrase; KIF5B, kinesin-1 heavy chain; NC, nucleocapsid; NPC, nuclear pore complex; Nup, nucleoporin; MARK2, microtubule affinity-regulating kinase 2; MEK1/2, mitogen-activated protein 1 and 2; MELK, maternal embryonic leucine zipper kinase; MTOC, microtubule-organizing centers; MxB, myxovirus resistance protein B; PDZD8, PDZ domain-containing 8 protein; PIC, pre-integration complex; RT, reverse transcriptase; RTC, reverse transcription complexes; TNPO3, transportin 3.

Early HIV-1 replication requires fusion of viral and cellular membranes that release a fullerene-shaped viral core structure, formed by a lattice of primarily hexameric capsid (CA) rings, into the cellular cytoplasm. The core contains positive-polarity single-strand viral genomic RNA and enzymes that synthesize viral double-strand DNA that is integrated into a host chromosome. Only HIV-1 that release intact cores into the cytoplasm are infectious [1], and disassembly of the core occurs by a regulated process called uncoating. For the purpose of this article, we define uncoating as any dissociation of CA from the viral core. However, the precise mechanism, timing, and location of uncoating is contentious. Recent live-cell single-particle tracking of infectious HIV-1 cores has provided unprecedented insight into uncoating kinetics [2]. One such study found that core integrity, measured by “leakiness” of a core-trapped fluorescent marker, was lost in the cytoplasm approximately 30 min after fusion (after first-strand transfer of reverse transcription) and that this was required for productive infection [3]. However, any such loss of core integrity must not allow innate cellular sensors, such as cyclic guanosine monophosphate–adenosine monophosphate synthase (cGAS), to identify and restrict retroviral DNA in the cytoplasm [4]. Another recent study found that a small amount of CA dissociates gradually from the core post-entry before the remaining core structure docks at the nuclear pore complex (NPC), leading to accelerated CA loss, nuclear entry, integration of viral DNA into host DNA, and productive infection [5]. As these two studies measure different events and are not mutually exclusive, it is our view, consistent with that expressed in the review by Francis and Melikyan [2], that HIV-1 uncoating may begin by the loss of core integrity and gradual CA dissociation in the cytoplasm, followed by CA-dependent nuclear docking and accelerated CA loss at the NPC. An alternative model of uncoating is that an intact core arrives at the NPC before uncoating [1]. Here, we discuss the host cell factors subverted by HIV-1 to regulate ordered uncoating in cells (Fig 1), with the caveat that the spatiotemporal specifics of where and when host factors interact with the core and affect CA uncoating requires further investigation.

## HIV-1 hijacks the cellular cytoskeleton for nuclear trafficking and successful uncoating

The cellular cytoskeleton is made up of filamentous actin, intermediate filaments, and microtubules, which function to maintain cell structural integrity and transport cargo within the cell. McDonald and colleagues used HIV-1 with green fluorescent protein (GFP) fused to the viral protein R (Vpr) to monitor HIV-1 trafficking from the cytoplasmic periphery to the nucleus. This revealed that replication complexes were associated with microtubules and accumulated near microtubule-organizing centers (MTOC) at the nuclear periphery [6]. In addition to facilitating cytoplasmic–nuclear trafficking of the core, the engagement of the host cytoskeleton by HIV-1 is important for regulated uncoating.

Treatment of cells with drugs called nocodazole (microtubule polymerization inhibitor) or ciliobrevin D (dynein inhibitor), as well as knockdown of dynein cytoplasmic 1 heavy chain 1 (DYNC1H1), dynein light chain 1 (DYNLL1), or the kinesin-1 heavy chain (KIF5B), all result in delayed HIV-1 uncoating in cells [7, 8]. Evidence shows, however, that kinesin-1 light chains are not involved in uncoating [9]. The core movement along microtubules involves CA interaction with the kinesin-1 adapter fasciculation and elongation factor zeta 1 (FEZ1) [10] and CA interaction with the dynein adaptor bicaudal D2 (BICD2) [11]. HIV-1 CA–nucleocapsid (NC) complexes bind to microtubule affinity-regulating kinase 2 (MARK2) for localized FEZ1 phosphorylation, which is required for FEZ1 interaction with kinesin-1 and stimulation of core trafficking and uncoating [9]. As the capsids of some other viruses directly bind to microtubule motor proteins [12], it is unclear why HIV-1 utilizes adaptor proteins rather than direct CA interaction with dyneins and kinesins. It is possible that HIV-1 has more regulatory capacity over the adaptor proteins rather than dyneins and kinesins directly, as shown, for example, by the phosphorylation of FEZ1, which may allow it to tightly regulate and balance microtubule transport and uncoating. Furthermore, DYNLL1 binds to the HIV-1 integrase (IN) protein [8], which subverts the potential issue of a bound CA uncoating and halting nuclear transport, as IN remains associated with HIV-1 complexes until DNA integration.

In addition, two cellular factors that support stable microtubules have been linked to core uncoating, without directly mediating cytoskeleton-dependent core trafficking. The diaphanous-related formins 1 and 2 (Dia1 and Dia2) are CA-binding proteins and important cytoskeleton regulatory proteins that stabilize microtubules in HIV-1 infected cells and promote uncoating and viral DNA synthesis [13]. PDZ domain-containing 8 protein (PDZD8) is a moesin-interacting protein that regulates microtubule stability [14] and also promotes HIV-1 infection by direct binding to CA–NC complexes and stabilization of HIV-1 cores [15].

The combined evidence highlights the importance of intact microtubules for regulated core uncoating. Lukic and colleagues speculate that uncoating may occur through a “tug-of-war” between dynein and kinesin motors bound to a single core, which may result in dissociation of CA as the core is pulled in opposite directions and/or that microtubule localization may allow favorable spatiotemporal positioning of the HIV-1 core in the cell for optimal uncoating kinetics [7].

## The dynamic ability of eEF1A to both promote core uncoating and stabilize CA associated with RTCs

Eukaryotic translation elongation factor 1A (eEF1A) is a highly abundant protein primarily involved in delivering aa-tRNAs to the ribosomes during translation elongation, and our work has shown that eEF1A also binds tightly and directly with HIV-1 reverse transcriptase (RT) and is important for HIV-1 reverse transcription [16]. We showed that a point mutation of a single strictly conserved HIV-1 RT surface-exposed residue, E300R, reduced HIV-1 RT interaction with eEF1A, resulting in a delay in reverse transcription and uncoating kinetics in cells [17]. A panel of RT mutations was used to show that uncoating kinetics were not solely dependent on reverse transcription kinetics but instead correlated with the mutant RT interaction with eEF1A [17]. While the mechanism is unclear, we speculate that there are two possibilities: 1) virion-associated eEF1A [18] stimulates post-entry uncoating or 2) eEF1A in an infected cell binds RT in a partially uncoated core and stimulates further downstream CA dissociation up to two hours post-infection (hpi). However, our earlier study found the level of CA in cell fractions containing reverse transcription complexes (RTCs) at four hpi was reduced when cells were treated with an eEF1A-binding drug called didemnin B, indicating eEF1A stabilizes CA association with the RTCs later in infection [16]. The link between the efficiency of HIV-1 reverse transcription and the kinetics of core uncoating [19] suggests that multiple host cofactors may work in concert to coregulate reverse transcription and uncoating, and this is likely to be a fruitful research area. It is notable that eEF1A is also a cytoskeletal regulator, and the uncoating regulators Dia1 and Dia2 have an eEF1A-binding site [20], but it is not known if an eEF1A interaction with Dia1/2 is important for HIV-1 uncoating.

## Two specific human PPIase proteins regulate uncoating by opposing mechanisms

Peptidyl-prolyl isomerases (PPIases) are involved in protein folding by isomerising *cis* and *trans* isomers of proline residues in polypeptides. PPIase A, also known as cyclophilin A (CypA), is a multifunctional CA-binding host protein that is packaged into HIV-1 virions [21]. Several studies have found that CypA stabilizes HIV-1 cores in vitro [22–24], and cryogenic electron microscopy (cryoEM) shows that it does this by binding two CA molecules from adjacent hexamers [24]. This enhanced core stability prevents premature uncoating of HIV-1 cores in human CD4 expressing T cells and enables productive infection [22], possibly by preventing premature innate sensing of retroviral DNA in the cytoplasm.

Another PPlase, Pin1, was found to promote uncoating *in vitro* and in human cells. Misumi and colleagues showed that Pin1 interacts with the phosphorylated Ser^16^-Pro^17^ CA motif, and HIV-1 with the S16A/P17A mutations had defective uncoating and reverse transcription, similar to wild-type HIV-1 in Pin1 knockdown cells [25]. A subsequent study identified that extracellular signal-regulated kinase 2 (ERK2) was incorporated into HIV-1 virions and phosphorylated the Ser^16^ CA motif during virion maturation [26]. Furthermore, treatment of HIV-1 producer cells with the mitogen-activated protein 1 and 2 (MEK1/2) inhibitor trametinib reduced levels of active phosphorylated ERK2 in the virion and delayed uncoating upon infection [27]. These studies combined indicate that virion-associated MEK1/2 activates virion-associated ERK2 by phosphorylation, and this is important for Ser^16^ phosphorylation during HIV-1 maturation, allowing cellular Pin1 to bind this motif and regulate uncoating and reverse transcription. Another cellular kinase, maternal embryonic leucine zipper kinase (MELK), was recently found to phosphorylate CA Ser^149^ in the cytoplasm to promote core uncoating [28].

## Human nucleus-associated proteins bind CA for nuclear import and final stages of CA dissociation

Evidence is emerging that a small amount of HIV-1 CA remains associated with the viral replication complex for post-nuclear entry stages of replication. Host cell proteins that bind CA near or within the nucleus are required for nuclear import and may also be involved in disassembly of the CA that remains associated. The CA remaining with the pre-integration complex (PIC) can interact with proteins of the nuclear pore complex nucleoporin 358 and 153 (Nup358 and Nup153), and this is important for nuclear import of the PIC and final stages of uncoating regulation. Specifically, Nup358 has been shown to facilitate uncoating in cooperation with kinesin-1 in the cytoplasm [29], and Nup153 has been shown to prolong CA association with PICs in the nucleus [30].

Cleavage and polyadenylation specificity factor 6 (CPSF6) binds to CA and stabilizes the HIV-1 core in the cytoplasm of infected cells [31]. Similarly to myxovirus resistance protein B (MxB) oligomers [32], this results in a hyperstable core with defective uncoating and infectivity. However, CPSF6 is normally localized to the nucleus by transportin 3 (TNPO3), allowing normal uncoating and permissiveness of HIV-1 [31]. This suggests that TNPO3 may stimulate HIV-1 CA uncoating indirectly by localizing CPSF6 to the nucleus, where it promotes nuclear entry and integration [33]. However, TNPO3 was also shown to stimulate HIV-1 CA uncoating *in vitro* and in cells and may be important for dissociation of the last remaining CA from the PIC in the nucleus before integration of the viral DNA into the host genomic DNA [23, 34].

## Perspectives

Research investigating the mechanisms of host cell factor facilitation of viral replication is intensifying to better understand how viruses with limited genomic capacities can successfully complete their complex replication processes, and this may translate to novel therapeutic targets in the future. It is becoming clearer that HIV-1 uncoating is a complex process that is regulated by several host cell proteins, and investigating the regulatory mechanisms of these host proteins may provide insight into the elusive specificities of uncoating spatiotemporal regulation. Therapies that block or enhance these virus–host interactions may cause premature or delayed uncoating and have potential to be a new addition to the combination antiretroviral therapy for HIV-1 patients.

## References

[1] Campbell EM, Hope TJ. HIV-1 capsid: the multifaceted key player in HIV-1 infection. Nature reviews Microbiology. 2015;13(8):471–83. 10.1038/nrmicro3503 26179359 PMC4876022

[2] Francis AC, Melikyan GB. Live-Cell Imaging of Early Steps of Single HIV-1 Infection. Viruses. 2018;10(5). 10.3390/v10050275 PMC5977268 29783762

[3] Mamede JI, Cianci GC, Anderson MR, Hope TJ. Early cytoplasmic uncoating is associated with infectivity of HIV-1. Proc Natl Acad Sci U S A. 2017;114(34):E7169–e78. 10.1073/pnas.1706245114 28784755 PMC5576815

[4] Gao D, Wu J, Wu YT, Du F, Aroh C, Yan N, et al Cyclic GMP-AMP synthase is an innate immune sensor of HIV and other retroviruses. Science. 2013;341(6148):903–6. 10.1126/science.1240933 23929945 PMC3860819

[5] Francis AC, Melikyan GB. Single HIV-1 Imaging Reveals Progression of Infection through CA-Dependent Steps of Docking at the Nuclear Pore, Uncoating, and Nuclear Transport. Cell Host Microbe. 2018;23(4):536–48.e6. 10.1016/j.chom.2018.03.009 29649444 PMC5901770

[6] McDonald D, Vodicka MA, Lucero G, Svitkina TM, Borisy GG, Emerman M, et al Visualization of the intracellular behavior of HIV in living cells. J Cell Biol. 2002;159(3):441–52. 10.1083/jcb.200203150 12417576 PMC2173076

[7] Lukic Z, Dharan A, Fricke T, Diaz-Griffero F, Campbell EM. HIV-1 uncoating is facilitated by dynein and kinesin 1. Journal of virology. 2014;88(23):13613–25. 10.1128/JVI.02219-14 25231297 PMC4248982

[8] Jayappa KD, Ao Z, Wang X, Mouland AJ, Shekhar S, Yang X, et al Human immunodeficiency virus type 1 employs the cellular dynein light chain 1 protein for reverse transcription through interaction with its integrase protein. J Virol. 2015;89(7):3497–511. 10.1128/JVI.03347-14 25568209 PMC4403391

[9] Malikov V, Naghavi MH. Localized Phosphorylation of a Kinesin-1 Adaptor by a Capsid-Associated Kinase Regulates HIV-1 Motility and Uncoating. Cell Rep. 2017;20(12):2792–9. 10.1016/j.celrep.2017.08.076 28930676 PMC5679262

[10] Malikov V, da Silva ES, Jovasevic V, Bennett G, de Souza Aranha Vieira DA, Schulte B, et al HIV-1 capsids bind and exploit the kinesin-1 adaptor FEZ1 for inward movement to the nucleus. Nature communications. 2015;6:6660 10.1038/ncomms7660 25818806 PMC4380233

[11] Dharan A, Opp S, Abdel-Rahim O, Keceli SK, Imam S, Diaz-Griffero F, et al Bicaudal D2 facilitates the cytoplasmic trafficking and nuclear import of HIV-1 genomes during infection. Proc Natl Acad Sci U S A. 2017. 10.1073/pnas.1712033114 PMC5740630 29180435

[12] Dodding MP, Way M. Coupling viruses to dynein and kinesin-1. EMBO J. 2011;30(17):3527–39. 10.1038/emboj.2011.283 21878994 PMC3181490

[13] Delaney MK, Malikov V, Chai Q, Zhao G, Naghavi MH. Distinct functions of diaphanous-related formins regulate HIV-1 uncoating and transport. Proc Natl Acad Sci U S A. 2017;114(33):E6932–e41. 10.1073/pnas.1700247114 28760985 PMC5565409

[14] Henning MS, Stiedl P, Barry DS, McMahon R, Morham SG, Walsh D, et al PDZD8 is a novel moesin-interacting cytoskeletal regulatory protein that suppresses infection by herpes simplex virus type 1. Virology. 2011;415(2):114–21. 10.1016/j.virol.2011.04.006 21549406

[15] Guth CA, Sodroski J. Contribution of PDZD8 to stabilization of the human immunodeficiency virus type 1 capsid. J Virol. 2014;88(9):4612–23. 10.1128/JVI.02945-13 24554657 PMC3993830

[16] Li D, Wei T, Rawle DJ, Qin F, Wang R, Soares DC, et al Specific Interaction between eEF1A and HIV RT Is Critical for HIV-1 Reverse Transcription and a Potential Anti-HIV Target. PLoS Pathog. 2015;11(12):e1005289 10.1371/journal.ppat.1005289 26624286 PMC4666417

[17] Rawle DJ, Li D, Swedberg JE, Wang L, Soares DC, Harrich D. HIV-1 Uncoating and Reverse Transcription Require eEF1A Binding to Surface-Exposed Acidic Residues of the Reverse Transcriptase Thumb Domain. MBio. 2018;9(2). 10.1128/mBio.00316-18 PMC5874916 29588400

[18] Cimarelli A, Luban J. Translation Elongation Factor 1-Alpha Interacts Specifically with the Human Immunodeficiency Virus Type 1 Gag Polyprotein. Journal of virology. 1999;73(7):5388–401. 10364286 10.1128/jvi.73.7.5388-5401.1999 PMC112595

[19] Hulme AE, Perez O, Hope TJ. Complementary assays reveal a relationship between HIV-1 uncoating and reverse transcription. Proceedings of the National Academy of Sciences of the United States of America. 2011;108(24):9975–80. 10.1073/pnas.1014522108 21628558 PMC3116424

[20] Peng J, Wallar BJ, Flanders A, Swiatek PJ, Alberts AS. Disruption of the Diaphanous-related formin Drf1 gene encoding mDia1 reveals a role for Drf3 as an effector for Cdc42. Curr Biol. 2003;13(7):534–45. 12676083 10.1016/s0960-9822(03)00170-2

[21] Ambrose Z, Aiken C. HIV-1 uncoating: connection to nuclear entry and regulation by host proteins. Virology. 2014;454–455:371–9. 10.1016/j.virol.2014.02.004 24559861 PMC3988234

[22] Li Y, Kar AK, Sodroski J. Target cell type-dependent modulation of human immunodeficiency virus type 1 capsid disassembly by cyclophilin A. J Virol. 2009;83(21):10951–62. 10.1128/JVI.00682-09 19656870 PMC2772774

[23] Shah VB, Shi J, Hout DR, Oztop I, Krishnan L, Ahn J, et al The host proteins transportin SR2/TNPO3 and cyclophilin A exert opposing effects on HIV-1 uncoating. J Virol. 2013;87(1):422–32. 10.1128/JVI.07177-11 23097435 PMC3536424

[24] Liu C, Perilla JR, Ning J, Lu M, Hou G, Ramalho R, et al Cyclophilin A stabilizes the HIV-1 capsid through a novel non-canonical binding site. Nat Commun. 2016;7:10714 10.1038/ncomms10714 26940118 PMC4785225

[25] Misumi S, Inoue M, Dochi T, Kishimoto N, Hasegawa N, Takamune N, et al Uncoating of human immunodeficiency virus type 1 requires prolyl isomerase Pin1. J Biol Chem. 2010;285(33):25185–95. 10.1074/jbc.M110.114256 20529865 PMC2919081

[26] Dochi T, Nakano T, Inoue M, Takamune N, Shoji S, Sano K, et al Phosphorylation of human immunodeficiency virus type 1 capsid protein at serine 16, required for peptidyl-prolyl isomerase-dependent uncoating, is mediated by virion-incorporated extracellular signal-regulated kinase 2. J Gen Virol. 2014;95(Pt 5):1156–66. 10.1099/vir.0.060053-0 24509437

[27] Dochi T, Akita A, Kishimoto N, Takamune N, Misumi S. Trametinib suppresses HIV-1 replication by interfering with the disassembly of human immunodeficiency virus type 1 capsid core. Biochem Biophys Res Commun. 2017. 10.1016/j.bbrc.2017.11.177 29197575

[28] Takeuchi H, Saito H, Noda T, Miyamoto T, Yoshinaga T. Phosphorylation of the HIV-1 capsid by MELK triggers uncoating to promote viral cDNA synthesis. PLoS Pathog. 2017;13(7):e1006441 10.1371/journal.ppat.1006441 28683086 PMC5500366

[29] Dharan A, Talley S, Tripathi A, Mamede JI, Majetschak M, Hope TJ. KIF5B and Nup358 Cooperatively Mediate the Nuclear Import of HIV-1 during Infection. PLoS Pathog. 2016;12(6):e1005700 10.1371/journal.ppat.1005700 27327622 PMC4915687

[30] Chen NY, Zhou L, Gane PJ, Opp S, Ball NJ, Nicastro G, et al HIV-1 capsid is involved in post-nuclear entry steps. Retrovirology. 2016;13:28 10.1186/s12977-016-0262-0 27107820 PMC4842275

[31] De Iaco A, Santoni F, Vannier A, Guipponi M, Antonarakis S, Luban J. TNPO3 protects HIV-1 replication from CPSF6-mediated capsid stabilization in the host cell cytoplasm. Retrovirology. 2013;10:20 10.1186/1742-4690-10-20 23414560 PMC3599327

[32] Fricke T, White TE, Schulte B, de Souza Aranha Vieira DA, Dharan A, Campbell EM, et al MxB binds to the HIV-1 core and prevents the uncoating process of HIV-1. Retrovirology. 2014;11:68 10.1186/s12977-014-0068-x 25123063 PMC4145229

[33] Chin CR, Perreira JM, Savidis G, Portmann JM, Aker AM, Feeley EM, et al Direct Visualization of HIV-1 Replication Intermediates Shows that Capsid and CPSF6 Modulate HIV-1 Intra-nuclear Invasion and Integration. Cell Rep. 2015;13(8):1717–31. 10.1016/j.celrep.2015.10.036 26586435 PMC5026322

[34] Zhou L, Sokolskaja E, Jolly C, James W, Cowley SA, Fassati A. Transportin 3 promotes a nuclear maturation step required for efficient HIV-1 integration. PLoS Pathog. 2011;7(8):e1002194 10.1371/journal.ppat.1002194 21901095 PMC3161976

